# “We are Scapegoats”: Front-Line Nursing Home Staff Experiences During the COVID-19 Pandemic

**DOI:** 10.1093/geroni/igaa057.3527

**Published:** 2020-12-16

**Authors:** Elizabeth White, Terrie Wetle, Ann Reddy, Rosa Baier

**Affiliations:** 1 Brown University School of Public Health, Providence, Rhode Island, United States; 2 Brown University, School of Public Health, Providence, Rhode Island, United States; 3 Brown University, Providence, Rhode Island, United States; 4 Center for Long-Term Care Quality & Innovation, Providence, Rhode Island, United States

## Abstract

The COVID-19 pandemic is an unprecedented challenge for nursing homes, where staff have faced rapidly evolving circumstances to care for a vulnerable resident population. To document these healthcare professionals’ experiences during the pandemic, we used social media and professional networks to disseminate an electronic survey with closed- and open-ended questions to a convenience sample of long-term care staff from May 11 through June 4, 2020. Four investigators identified themes from qualitative responses for 152 nursing home staff respondents from 32 states. Key themes included: constraints on personal protective equipment (PPE) and testing; burdensome regulations and guidance; concern for self, family, and residents; workforce burnout; organizational communication and teamwork; and public lack of recognition. Respondents described ongoing constraints on testing, and reliance on crisis standards for extended use and reuse of PPE. Administrators discussed implementing sometimes confusing or contradictory guidance from numerous agencies. Direct-care staff expressed fears of infecting themselves and their families, and expressed empathy and concern for their residents. They described burnout due to increased workloads and the emotional burden of caring for residents facing isolation, illness, and death. Respondents cited the presence or lack of organizational communication and teamwork as factors influencing their ability to work under challenging circumstances. They also described the demoralizing impact of negative media coverage of nursing homes, contrasting this with the heroic public recognition given to hospital staff. These challenges added significant burden to an already strained workforce and are likely to contribute to increased burnout, turnover, and staff shortages in the long-term.

